# A ‘modified de Gramont’ regimen of fluorouracil, alone and with oxaliplatin, for advanced colorectal cancer

**DOI:** 10.1038/sj.bjc.6600467

**Published:** 2002-08-12

**Authors:** S L Cheeseman, S P Joel, J D Chester, G Wilson, J T Dent, F J Richards, M T Seymour

**Affiliations:** Cancer Research UK Clinical Centre in Leeds, Cookridge Hospital, Leeds LS16 6QB, UK; Barry Reed Oncology Laboratory, St Bartholomew's Hospital, London EC1A 7BE, UK

**Keywords:** fluorouracil, oxaliplatin, colorectal carcinoma

## Abstract

The standard de Gramont (dG) regimen of fortnightly leucovorin, bolus fluorouracil and 22-h infusion of fluorouracil, d1+2, and the same regimen plus oxaliplatin, are effective but also cumbersome. We therefore present simplified ‘Modified de Gramont’ (MdG) regimens. Forty-six advanced gastrointestinal cancer patients entered a dose-exploring study of MdG, including an expanded cohort of colorectal cancer patients at optimum dose. Treatment (fortnightly) comprised: 2-h i.v.i. leucovorin (350 mg *d,l-*LV or 175 mg *l-*LV, not adjusted for patient surface area); bolus fluorouracil (400 mg m^−2^), then ambulatory 46-h fluorouracil infusion (2000–3600 mg m^−2^, cohort escalation). Subsequently, 62 colorectal patients (25 unpretreated; 37 fluorouracil-resistant) received MdG plus oxaliplatin (OxMdG) 85 mg m^−2^. Fluorouracil pharmacokinetics during MdG were compared with dG. The optimum fluorouracil doses for MdG alone were determined as 400 mg m^−2^ bolus + 2800 mg m^−2^ 46-h infusion. A lower dose of 400 mg m^−2^ bolus + 2400 mg m^−2^ infusion which, like dG produces minimal toxicity, was chosen for the OxMdG combination. Fluorouracil exposure (AUC_0–48 h_) at this lower dose is equivalent to dG. With OxMdG, grade 3–4 toxicity was rare (neutropenia 2.8% cycles; vomiting or diarrhoea <1% cycles), but despite this there were two infection-associated deaths. Oxaliplatin was omitted for cumulative neurotoxicity in 17 out of 62 patients. Objective responses in colorectal cancer patients were: 1st-line MdG (22 assessable): PR=36%, NC=32%, PD=32%. 1st-line OxMdG (24 assessable): CR/PR=72%; NC=20%; PD=8%; 2nd line OxMdG (34 assessable): PR=12%; NC=38%; PD=50%. MdG and OxMdG are convenient and well-tolerated. OxMdG was particularly active as 1st-line treatment of advanced colorectal cancer. Both regimens are being further evaluated in the current UK MRC phase III trial.

*British Journal of Cancer* (2002) **87**, 393–399. doi:10.1038/sj.bjc.6600467 www.bjcancer.com

© 2002 Cancer Research UK

## 

The de Gramont regimen (dG), also known as ‘LV5FU2’, is one of several standard methods for administration of 5-fluorouracil (FU) and leucovorin (LV). It involves a 2-h infusion of LV (200 mg m^−2^), bolus injection of FU (400 mg m^−2^), then 22-h infusion of FU (600 mg m^−2^), with the same sequence repeated on the second day, repeated fortnightly (de Gramont *et al*, 1988). It was compared with the Mayo Clinic 5-day bolus FU/LV regimen in a 448-patient randomised trial, and showed a better response rate (32.6% *vs* 14.4%; *P*=0.0004), and median progression-free survival (27.6 *vs* 22 weeks; *P*<0.0012) with significantly reduced rates of diarrhoea, mucositis and neutropenia; however, overall survival was not significantly improved (de Gramont *et al*, 1997). Following this trial dG was adopted as a standard therapy option by many oncologists, especially in France and the UK.

Its low toxicity profile makes dG a good basis for combination chemotherapy. Pivotal trials of the design ‘dG±new agent’ have been performed in first-line therapy of metastatic colorectal cancer using oxaliplatin (de Gramont *et al*, 2000) or irinotecan (Douillard *et al*, 2000), in each case producing a high response rate and good safety profile. Similar trials are now ongoing in the adjuvant setting.

Although dG can be administered on an ambulatory, out-patient basis, many units find it more convenient to admit patients. This, together with the high dose of LV, and a labour-intensive administration schedule, place high demands on healthcare resources (Ross *et al*, 1998). Furthermore, repeated hospital visits or admissions during dG may detract from the benefits of its low toxicity profile.

Along with others (see Discussion), we reasoned that it would be possible to modify the dG regimen, reducing its costs and making it universally applicable as an outpatient regimen, whilst exploring higher FU dose-intensity which might further improve its efficacy. The ‘Modified de Gramont’ (MdG) regimens incorporate two main changes:

1. LV at a flat dose (350 mg *d,l-* or 175 mg *l-*LV, not adjusted for patient surface area), and on day 1 only. There is no evidence for a dose-response effect with LV (Ychou *et al*, 1998), so surface-area dosing is unjustified. Prolonged (>24 h) high levels of plasma reduced folate metabolites are obtained after LV infusion at this dose.
2. FU is given as a single 400 mg m^−2^ bolus, then high dose-rate 46-h infusion. This avoids the need for day 2 ward attendance, reduces nurse and pharmacist time and makes the schedule more suitable for the out-patient setting.

First, a cohort dose-escalation study was performed to determine the optimum FU 46-h infusion dose. The aim was to find (a) the dose producing equivalent low toxicity to dG, as a basis for combination chemotherapy regimens, and (b) the FU dose most suitable for further study when MdG is used alone. An expanded cohort of patients with unpretreated metastatic colorectal cancer was studied at this latter dose to confirm the activity of the new MdG regimen. The lower dose was then combined with oxaliplatin (‘OxMdG’) and studied in patients with FU-resistant or unpretreated metastatic colorectal cancer.

FU pharmacokinetics are non-linear, so the higher dose-intensities of FU commonly achieved with infusional treatment are no guarantee of increased tumour exposure to the drug. We therefore performed a pharmacokinetic study to assess FU exposure (area under the plasma concentration-time curve, AUC) during the new MdG regimens compared with dG.

## PATIENTS AND METHODS

### Patients

The studies involved a total of 108 patients (see Table 1

**Table 1:**
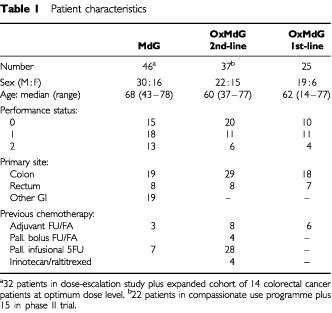
Patient characteristics

). Appropriate Institutional and Local Ethics approval was obtained, and all patients gave written informed consent.

#### MdG (46 patients)

To establish the optimum 46-h FU infusion dose, an escalation study was performed in 32 patients with metastatic adenocarcinoma of any gastrointestinal primary origin. Eligibility criteria were WHO performance status 0–2; bilirubin <50 μmol l^−1^; ALP and transaminases <3×upper limit of normal; WBC >3×10^9^ l^−1^; neutrophils >1.5×10^9^ l^−1^ and platelets >100× 10^9^ l^−1^. Women of child bearing potential were required to use contraception. Previous chemotherapy for metastatic disease was permitted. Following this, MdG at the 400 mg m^−2^ bolus + 2800 mg m^−2^ 46-h infusion dose level was adopted in our institution as a standard therapy option for patients with metastatic colorectal cancer fulfilling the same eligibility criteria. Toxicity and response data were collected for a further 14 patients, giving a total of 22 patients with metastatic colorectal cancer treated at that dose level for analysis.

#### OxMdG (62 patients)

Initially, 22 patients with FU-resistant colorectal cancer were treated with OxMdG in a named-patient compassionate-use programme. Patients had to have inoperable, histologically confirmed colorectal adenocarcinoma with progression during or soon after prior chemotherapy . The same general fitness and organ function criteria were used as for MdG, with the addition of GFR >60 ml min^−1^. From May 1999, the use of OxMdG in our institution was expanded to a formal phase II trial including a further fifteen 2nd-line patients (bringing the total to 37) and 25 patients with unpretreated metastatic colorectal cancer. The same eligibility criteria applied.

### Treatment

Single-lumen venous access was established using a subcutaneous port or Hickman line. Prophylactic warfarin was given, 10 mg on the day of line insertion then 1 mg daily.

**Figure 1:**
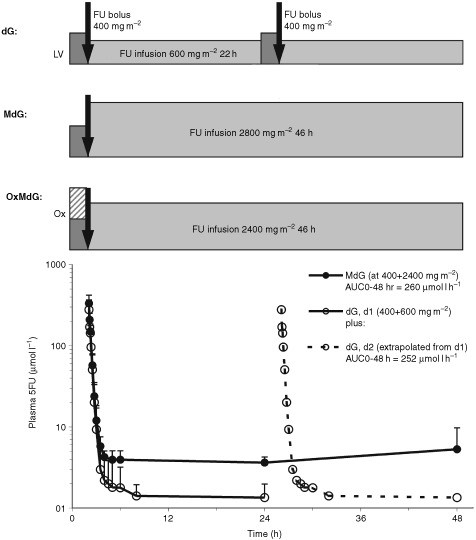
dG, MdG and OxMdG. Plasma FU levels are shown for nine patients receiving dG (day 2 data extrapolated from day 1) and in 10 receiving MdG, at the lower dose of 400 mg m^−2^ bolus+2400 mg m^−2^ 46 h infusion as used in the OxMdG schedule.

MdG comprised: fixed dose *d,l-*LV 350 mg (or *l-*LV, 175 mg) as a 2-h i.v. infusion; then FU i.v. bolus over 5 min; then 46-h FU infusion (see Figure 1

). Treatment was repeated every 14 days. MdG was given without routine prophylactic anti-emetics or anti-diarrhoeals, but patients were supplied with standard doses of oral metoclopramide and loperamide with written instructions on their use. The 46-h FU infusion was delivered using a disposable elastomeric pump (Baxter LV5®). After the infusion, the line was flushed by the patient's community nurse. Hickman lines were flushed weekly between treatments. For patients without central venous access, the infusion was given via a peripheral cannula, in hospital, until access has been established.

OxMdG was preceded by i.v. dexamethasone 8 mg and granisetron 1 mg, and followed by oral dexamethasone (4 mg t.d.s. on day 2; b.d. on day 3 and o.d. on day 4). Oxaliplatin 85 mg m^−2^ was given concurrently with LV, via a Y-connector, during the first 2 h. Each drug was diluted in 250 ml 5% dextrose, and care was taken to avoid mixing oxaliplatin with saline. Thereafter, OxMdG was administered in the same way as MdG.

### Chemotherapy starting-dose and adjustments

LV was not adjusted for toxicity. The starting-dose for the 5-min bolus FU was 400 mg m^−2^ in all patients, both for MdG and OxMdG. The dose of 46-h FU infusion was studied by dose escalation in the first 32 patients receiving MdG. Five dose-levels were investigated, 2000–3600 mg m^−2^ (Table 2

**Table 2:**
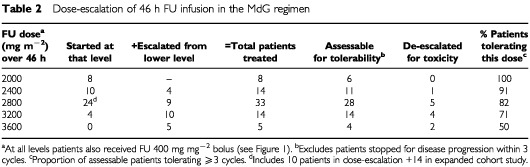
Dose-escalation of 46 h FU infusion in the MdG regimen

). Patients were evaluated for toxicity after each cycle (NCI Common Toxicity Criteria v2.0). After cycle 4, intra-patient dose-escalation to the next level was permitted providing there had been no acute toxicity greater than CTC grade 2, or persistent grade 2 toxicity. The aim was to establish the dose at which treatment could continue indefinitely, therefore persistent symptomatic grade 2 toxicity was taken as an indication for dose de-escalation.

After completion of the dose-escalation study, the 46 h FU infusion dose was set at 2800 mg m^−2^ for patients receiving MdG alone, and 2400 mg m^−2^ for patients receiving OxMdG. These doses were not escalated thereafter. As before, toxicity was scored by the research nurse at the start of each cycle of chemotherapy. For day 1 WBC, ANC or platelet count of grade ⩾2, or for non-haematological toxicity of grade ⩾2 despite symptomatic measures, treatment was delayed 1 week. If a 2-week delay was required, or two separate delays of 1 week, subsequent chemotherapy doses (FU bolus; FU infusion; oxaliplatin, but not LV) were reduced by 20%.

Neurosensory toxicity was scored carefully in patients receiving OxMdG. Oxaliplatin was not adjusted for temporary cold-induced dysaethesia, but was omitted from the regimen for peripheral sensory neuropathy producing pain, numbness or loss of function (NCI CTC grade 3), if it persisted between chemotherapy cycles. Oxaliplatin was re-introduced if all symptoms of neuropathy resolved.

Treatment duration was not fixed. Chemotherapy was discontinued at disease progression, but treatment breaks were discussed with patients whose disease remained controlled after 12 cycles, resuming the same regimen at a later date.

### Evaluation of response and duration of treatment

Blood tests and clinical evaluation were performed twice-weekly, tumour marker assays 4-weekly and imaging (CT scanning±other tests) 12-weekly. Initially, response to chemotherapy was scored using WHO criteria, but after seeing unexpectedly high activity in the OxMdG first-line therapy cohort, all case notes and scans in this group were externally reviewed and scored using RECIST criteria (Therasse *et al*, 2000) by an independent oncologist and radiologist.

### Pharmacokinetics

Ten patients receiving OxMdG also participated in a study of FU pharmacokinetics, in which samples were taken during one cycle of OxMdG and one paired cycle of MdG alone (at the 2400 mg m^−2^ 46-h FU infusion dose). Blood was taken at 10 intervals out to 4 h, and at 24 and 46 h. Samples were cold-spun and the plasma frozen immediately after collection. Plasma 5-FU was determined by HPLC analysis (Seymour *et al*, 1994). The trapezoidal method was used to calculate FU AUC_0–48 h._ This study is reported elsewhere and shows that oxaliplatin does not significantly affect the plasma pharmacokinetics of FU (Joel *et al*, 2000).

For the current study, data for these 10 patients during MdG are compared with historical control data from nine patients previously studied using the same sampling and analytical techniques, in the same laboratory, during treatment with dG. Patients receiving dG underwent pharmacokinetic sampling during the first 24-h period, and these data have been extrapolated to estimate the FU AUC_0–48 h_. This assumes approximately equal FU pharmacokinetics on days 1 and 2 of the standard dG regimen.

## RESULTS

### Dose escalation and tolerability of MdG

Data for all 46 patients receiving MdG is summarised in Table 2. In general, MdG is a well-tolerated and practicable regimen. The dose-limiting side-effects in the short-term are gastrointestinal (diarrhoea, mucositis; nausea) and, after longer treatment, dermatological (hand/foot dermatitis). There were no episodes of grade ⩾3 haematological toxicity, even at the higher FU doses.

At lower dose levels, toxicity was confined to grade 2 nausea, diarrhoea, stomatitis or lethargy. One patient was de-escalated from 2400 to 2000 mg m^−2^ for grade 4 diarrhoea, but with this exception 2400 mg m^−2^ produced minimal toxicity, of a degree similar to the standard dG regimen.

At 2800 mg m^−2^, 5 out of 22 assessable patients eventually required de-escalation, in one case after grade 3 nausea but in all other cases for persistent grade 2 toxicity (nausea; diarrhoea; stomatitis; dermatitis). This degree of toxicity is higher than is seen with the standard dG regimen, but still less than conventional MTD, and this dose level was selected as the optimum starting dose for future use of MdG alone. Table 3

**Table 3:**
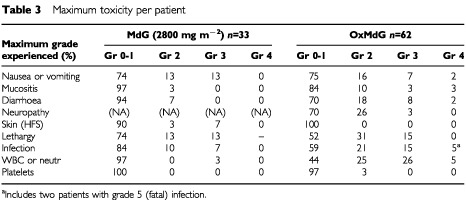
Maximum toxicity per patient

shows the maximum toxicity per patient for the 33 patients who received MdG at this dose level in either the dose-escalation study or the extended cohort.

Some patients are able to tolerate higher FU doses. At 3200 mg m^−2^, 71% patients were able to tolerate three or more cycles without dose reduction. However, grade 2 toxicities were frequent and led to dose reductions in 50% of patients after six cycles, so this dose was not selected for further study. No patients were entered at 3600 mg m^−2^ but of the five patients escalated to this level only two tolerated it for >three cycles. There were no treatment-related deaths at any level.

### Tolerability of OxMdG

The 62 OxMdG patients received a total of 778 treatment cycles. Serious adverse event (SAE) and maximum overall toxicity data are presented for all cycles in all patients (Table 3). Toxicity-per-cycle data is given only for the first six cycles, in patients on the formal phase II trial, to avoid bias from dose reductions and differing data collection methods (Table 4

**Table 4:**
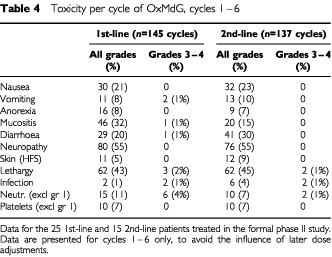
Toxicity per cycle of OxMdG, cycles 1–6

).

The toxicity profile was similar in first-line and second-line patients. Main toxicities were sensory neuropathy, lethargy, diarrhoea, nausea and neutropenia. However these rarely exceeded grade 2 (Table 4). In three patients oxaliplatin was omitted from the regimen after recurrent myelosuppression (neutropenia and/or thrombocytopenia) despite appropriate dose reductions. Mild sensory neuropathy was very common (55% of cycles). Among the 25 first-line patients in the formal phase II trial, nine required the omission of oxaliplatin for neurotoxicity at some point prior to treatment failure (median, 12 cycles). In FU-resistant patients, since disease progression on treatment occurred earlier, fewer (5 out of 37) required omission of oxaliplatin for cumulative toxicity.

Despite the generally favourable toxicity profile, two deaths were related to treatment (3.2% patients). One patient, after eight uneventful previous cycles, became progressively unwell over 48 h without seeking help, then was admitted urgently to the nearest hospital with neutropenia and respiratory failure, and died within a few hours. A second patient did not have neutropenia but succumbed with overwhelming methicillin-resistant *Staphylococcus aureus* (MRSA) septicaemia related to the central venous catheter.

### Anti-tumour efficacy

The MdG dose-escalation study was not designed to measure efficacy, but eight patients in the 2800 mg m^−2^ infusion cohort had unpretreated assessable advanced colorectal cancer. A further 14 colorectal patients were then entered at that level (without escalation) to give a total of 22 patients for a preliminary efficacy analysis (Table 5

**Table 5:**
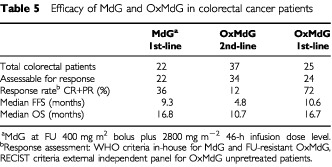
Efficacy of MdG and OxMdG in colorectal cancer patients

). Among these 22 patients, WHO PR was seen in eight (36%), with MR or SD for >12 weeks in a further seven (32%). Median failure-free survival (FFS) was 9.3 months. Following MdG, 15 of the 22 patients went on to receive second (±third) line chemotherapy and one responder underwent curative liver resection. Median overall survival (OS) from starting MdG is 16.8 months.

Tumour response was a formal endpoint of the OxMdG studies (Table 5). Thirty-four of the 37 second-line patients were assessable for response. Four patients achieved a confirmed partial response (12%), one patient an unconfirmed partial response (3%) and twelve (35%) had stable disease for at least 12 weeks. Median FFS was 4.8 months and median OS 10.7 months from the time of starting OxMdG.

For the 1st-line OxMdG cohort, all case-notes and scans were reviewed and scored for response using RECIST criteria, by an independent oncologist and radiologist appointed by ICRF. One patient, with disease seen only laparoscopically, was not assessable. One patient (4%) achieved a complete response; confirmed partial responses were seen in 17 (68%); unconfirmed (by a second scan) partial responses were seen in a further two patients (8%). In three patients (12%) disease remained stable for at least 12 weeks. Two patients progressed, in both cases after two cycles. The overall response rate (CR + confirmed PR) was 72%. Median failure-free survival is 10.6 months (range 0.9–24.7) and median overall survival is 16.7 months (range 2.0–26.7+).

Three patients with initially inoperable metastases underwent liver surgery after responding to OxMdG. Two have since relapsed, and one responded to re-challenge with the same regimen. One patient with mediastinal disease underwent consolidation mediastinal radiotherapy and remains disease-free 1 year later.

### Pharmacokinetics

Figure 1 and Table 6

**Table 6:**
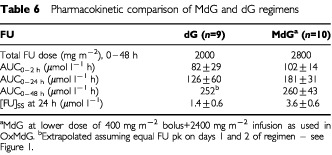
Pharmacokinetic comparison of MdG and dG regimens

show FU plasma profiles during the standard dG regimen, or MdG (at the lower, 2400 mg m^−2^ FU infusion dose level as used in OxMdG). The total area under the FU concentration-time curve during the regimen (AUC_0–48 h_) is similar for the two regimens (Table 6). This is consistent with the clinical finding, during the dose-escalation study, that despite its higher FU-dose intensity, MdG at this dose has the same minimal toxicity as standard dG.

## DISCUSSION

Fluoropyrimidines remain our best ‘single agent’ for colorectal cancer, and the basis of combination therapy. The optimum fluoropyrimidine therapy may continue to be debated, but de Gramont's LV5FU2 regimen (dG) is a strong contender, with a good track record of efficacy and low toxicity in large phase III randomised trials (de Gramont *et al*, 1997; Douillard *et al*, 2000; Maughan *et al*, 2002).

New cytotoxic drugs for colorectal cancer have brought the welcome need to develop safe, effective combination regimens. In Europe, dG has been combined with oxaliplatin and with irinotecan, in each case leading to successful phase III trials in advanced disease. In the first trial, 210 patients received oxaliplatin+dG, grade ⩾3 toxicities were frequent but manageable and one toxic death (<1%) was reported (de Gramont *et al*, 2000). In the second, 145 patients received irinotecan+dG, again with significant but manageable grade ⩾3 toxicity, and with one (<1%) toxic death (Douillard *et al*, 2000). Both regimens are now being assessed as adjuvant therapy, and whilst full data are awaited, interim toxicity reports are reassuring (Rothenberg *et al*, 2001). But these regimens are cumbersome for patients and, in this common disease, place formidable financial and human demands on healthcare resources.

Meanwhile, other groups based their combinations on bolus FU/LV regimens. A phase III trial in advanced disease compared bolus irinotecan, FU and LV, given weekly for 4 weeks out of 6 (IFL), against single-agent irinotecan or a FU/LV control: the combination treatment gave superior response rate and survival, with broadly similar rates of toxicity (Saltz *et al*, 2000). On this basis, IFL became standard care for metastatic colorectal cancer in USA and entered adjuvant trials, whilst other bolus FU-based combinations were evaluated in advanced disease. However, the toxicity of these combinations soon became cause for concern. Two novel schedules, involving irinotecan or oxaliplatin on day 1 with bolus FU/LV on days 2–5, were withdrawn from the NCCTG N9741 after excessive gastrointestinal and haematological toxicity, with a total of 10 toxic deaths among the first 108 patients treated (Morton *et al*, 2001). Subsequently, excess mortality during treatment with the IFL regimen in both N9741 and the CALGB C89803 adjuvant trial led to interruption of both these trials (Rothenberg *et al*, 2001; Sargent *et al*, 2001), although after a further review of all available toxicity data the US Oncology Drugs Advisory Committee decided not to remove the IFL regimen from the irinotecan product label (http://www.fda.gov/ohrms/dockets/ac/01/ transcripts/3815t2.rtf).

This leaves a clear need to develop regimens offering the good efficacy and safety profile of the dG-based combinations, but more convenient for patients and less demanding of healthcare resources. One approach is to explore the use of oral FU; the other, as used here, is to redesign and improve the FU infusion schedules. MdG preserves the main elements of dG: dose-intensive exposure to FU with LV for 48 h every 2 weeks, with minimal haematological gastrointestinal toxicity. MdG has not been compared with dG in a randomised trial; to demonstrate equivalence with reasonable confidence would require at least 1000 patients. However, two factors lend confidence to it: firstly, its activity during this pilot study was at least as good as expected with dG; secondly, the pharmacokinetic analysis shows equivalence in FU exposure (AUC_0–48 h_) for dG and the lower MdG dose used in the OxMdG schedule.

Similar but more dose-intensive MdG-like schedules have been developed by the French oncology group, GERCOR. Their simplified bimonthly regimen of FU and LV is similar to MdG but has approximately double the dose of *l-*LV (200 mg m^−2^, instead of 175 mg unadjusted for surface area), and includes individual titration of the FU dose up to 3600 mg m^−2^, which may potentially optimise 5-FU exposure, although the criteria for dose titration are not defined (Tournigand *et al*, 1998). In addition to these differences, the equivalent oxaliplatin combination, ‘FOLFOX6’, differs from OxMdG in using a higher dose of oxaliplatin (100 mg m^−2^ instead of 85 mg m^−2^) (Maindrault-Goebel *et al*, 1999). An even higher dose-intensity regimen, FOLFOX7, uses oxaliplatin at 130 mg m^−2^ fortnightly (Maindrault-Goebel *et al*, 2001).

It is not yet known whether these dose-intensive schedules, which may be used for only short treatment durations because of cumulative oxaliplatin neurotoxicity and carry a higher risk of myelotoxicity, have any advantage over the less dose-intensive OxMdG. In a preliminary report of a randomised trial examining the sequencing of oxaliplatin and irinotecan combinations, FOLFOX6 produced a response rate of 56% with median TTF of 8.9 months among 109 unpretreated patients (Tournigand *et al*, 2001), which does not suggest superior efficacy to OxMdG (see Table 5). Conversely, a comparison of response rates and survival in sequential second-line phase II studies from GERCOR has been reported as suggestive of an oxaliplatin dose-intensity effect (Maindrault-Goebel *et al*, 2000), and OxMdG appeared to perform relatively less well in our second-line cohort. Perhaps this serves to underline the unreliability of comparisons between different phase II studies; a true dose-intensity benefit will only be demonstrated with a prospective randomised trial.

Our group has also piloted an irinotecan+MdG combination, ‘IrMdG’, which includes irinotecan 180 mg m^−2^, with encouraging results (Ledermann *et al*, 2001). As with OxMdG, IrMdG is less dose-intensive of *l-*LV and FU than the equivalent GERCOR combination, FOLFIRI (Andre *et al*, 1999).

Importantly, compared with the original dG and FOLFOX4 regimens, the simple administration schedules of these ‘MdG’ regimens reduce patient and nurse time, and ensure that all patients may be treated on an outpatient basis with only one visit to the hospital every 2 weeks. In addition we found that these regimens helped foster close relationships between patient, Community Nurse and Oncology Unit, which in turn improved monitoring of toxicity during treatment and palliative care after eventual disease progression.

The regimens are well tolerated. Grade 3 and 4 toxicity is uncommon, even with OxMdG. Despite this, there were two unexpected deaths during OxMdG treatment (3.2%), underlining the need for rigorous patient and staff education. One of the deaths was due to overwhelming MRSA septicaemia, without neutropenia, attributable to the venous access device; the other was due to neutropenic sepsis managed too late and at a non-oncology hospital; both were potentially avoidable.

The efficacy of OxMdG was particularly encouraging in the cohort of unpretreated patients: 72% confirmed PR/CR, scored by an independently appointed external team. The 95% c.i. for this response rate (50–88%) is consistent with the response rates for similar regimens in two industry-run trials: FOLFOX4 gave responses in 50% of 210 patients, 95% c.i. 42–58% (de Gramont *et al*, 2000), and FOLFOX6 in 56% of 109, 95% c.i. 46–66% (Tournigand *et al*, 2001).

Equally noteworthy is the long overall survival observed in patients treated with MdG alone as first-line therapy. Sixty-eight percent of these patients received second-line and subsequent treatments after MdG; this raises the issue of whether patients are better served by first-line combination therapy or, as in this group, by maximising the period of disease control with an optimum FU/LV regimen before introducing other drugs. This question is being addressed in the current UK Medical Research Council phase III trial ‘Fluorouracil, Oxaliplatin and CPT-11: Use and Sequencing’ (FOCUS), which uses the MdG, OxMdG and IrMdG regimens as described here.

FOCUS is also an opportunity to evaluate the safety of OxMdG in the multicentre setting. Rigorous centre accreditation criteria are used, and adverse events are closely monitored. To date the record is good: with over 150 patients treated with OxMdG so far, there are no definite treatment-related deaths, and close vigilance continues.
